# Biocontrol potential of endophytic *Bacillus subtilis* A9 against rot disease of *Morchella esculenta*

**DOI:** 10.3389/fmicb.2024.1388669

**Published:** 2024-05-30

**Authors:** Xue Chen, Yin Zhang, ShengQian Chao, LiLi Song, GuoGan Wu, Yu Sun, YiFan Chen, BeiBei Lv

**Affiliations:** ^1^Biotechnology Research Institute, Key Laboratory of Agricultural Genetics and Breeding, Shanghai Academy of Agricultural Sciences, Shanghai, China; ^2^Key Laboratory for Safety Assessment (Environment) of Agricultural Genetically Modified Organisms, Ministry of Agriculture and Rural Affairs, P.R, Shanghai, China; ^3^Shanghai Professional Technology Service Platform of Agricultural Biosafety Evaluation and Testing, Shanghai Academy of Agricultural Sciences, Shanghai, China; ^4^Shanghai Co-Elite Agricultural Sci-Tech (Group) Co., Ltd., Shanghai, China; ^5^CIMMYT-China Specialty Maize Research Center, Shanghai, China

**Keywords:** *Morchella esculenta*, fungal disease, transcriptome analysis, *Lecanicillium aphanocladii*, biological control

## Abstract

**Introduction:**

*Morchella esculenta* is a popular edible fungus with high economic and nutritional value. However, the rot disease caused by *Lecanicillium aphanocladii*, pose a serious threat to the quality and yield of *M. esculenta*. Biological control is one of the effective ways to control fungal diseases.

**Methods and results:**

In this study, an effective endophytic *B. subtilis* A9 for the control of *M. esculenta* rot disease was screened, and its biocontrol mechanism was studied by transcriptome analysis. In total, 122 strains of endophytic bacteria from *M. esculenta*, of which the antagonistic effect of *Bacillus subtilis* A9 on *L. aphanocladii* G1 reached 72.2% *in vitro* tests. Biological characteristics and genomic features of *B. subtilis* A9 were analyzed, and key antibiotic gene clusters were detected. Scanning electron microscope (SEM) observation showed that *B. subtilis* A9 affected the mycelium and spores of *L. aphanocladii* G1. In field experiments, the biological control effect of *B. subtilis* A9 reached to 62.5%. Furthermore, the transcritome profiling provides evidence of *B. subtilis* A9 bicontrol at the molecular level. A total of 1,246 differentially expressed genes (DEGs) were identified between the treatment and control group. Gene Ontology (GO) enrichment analysis showed that a large number of DEGs were related to antioxidant activity related. Kyoto Encyclopedia of Genes and Genomes (KEGG) enrichment analysis showed that the main pathways were Nitrogen metabolism, Pentose Phosphate Pathway (PPP) and Mitogen-Activated Protein Kinases (MAPK) signal pathway. Among them, some important genes such as carbonic anhydrase CA (H6S33_007248), catalase CAT (H6S33_001409), tRNA dihydrouridine synthase DusB (H6S33_001297) and NAD(P)-binding protein NAD(P) BP (H6S33_000823) were found. Furthermore, *B. subtilis* A9 considerably enhanced the *M. esculenta* activity of Polyphenol oxidase (POD), Superoxide dismutase (SOD), Phenylal anineammonia lyase (PAL) and Catalase (CAT).

**Conclusion:**

This study presents the innovative utilization of *B. subtilis* A9, for effectively controlling *M. esculenta* rot disease. This will lay a foundation for biological control in *Morchella*, which may lead to the improvement of new biocontrol agents for production.

## Graphical abstract

Flow chart of experimental procedures. (Created with MedPeer, www.medpeer.cn).

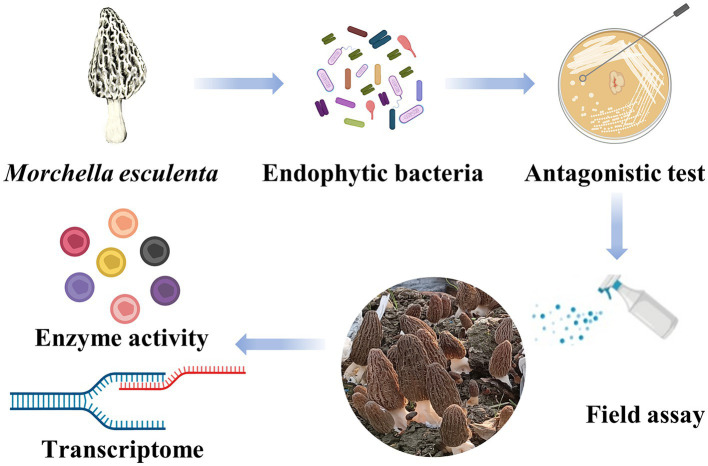

## 1 Introduction

As one of the most popular edible mushrooms, morel mushrooms are prized in many culinary applications for their rich aroma, savory taste, and crisp-tender flesh texture (Desjarden and Phillips, 1992; Du and Yang, 2021). Morel mushrooms belong to the *Morchella* genus of the *Morchellaceae* family of the *Ascomycota*. The prevalent cultivated species is *Morchella sextelata*, which has good commercial characteristics for fresh mushrooms. *M. sextelata* has recently been artificially cultivated in China and comprises >90% of the total area of *Morchella* cultivation (Deng et al., 2021; Yu et al., 2021). The fruiting bodies of morels are characterized by a unique conic head, with convex and concave folding on the surface (Tietel and Masaphy, 2018; Wu et al., 2021). *M. esculenta* contains all of the important nutrients, including carbohydrates, polyunsaturated fatty acids, and secondary metabolites, such as phenolic compounds. Its cultured mycelium is extensively used as a flavoring agent (Heleno et al., 2013). *M. esculenta* not only has a unique flavor and rich nutrition, but current scientific research has confirmed that it has antioxdant, anti-inflammatory, and immunostimulatory properties of medicinal value (Tietel and Masaphy, 2018; Sunil and Xu, 2022; Xu et al., 2022). Mushroom polysaccharides are used as anticancer drugs, including as treatments for skin and liver cancer. Morel polysaccharides also have possible uses for treatment of inflammatory diseases, and its lipopolysaccharides were shown to have potential to regulate the immune system by inhibiting the production of nitric oxide in macrophages. They are undeveloped sources of natural products and have great potential in the medicine and nutrition industries (Huang et al., 2011; Hu et al., 2012; Cai et al., 2018; Badshah et al., 2021). Therefore, the economic value of *M. esculenta* is very high, and market demand is constantly growing. However, the natural output of wild *M. esculenta* is limited, and the artificial cultivation technology of *Morchella* is not perfect. Thus, *M. esculenta* is in short supply (Liu W. et al., 2023).

Fungal diseases are important factors that seriously threaten the cultivation and growth of *Morchella* and are even more harmful than insect pests. Approximately 25% of the cultivation area has suffered annually from fungal diseases (He et al., 2017; Shi et al., 2022; Liu Z. et al., 2023). Currently, reported morel fungal diseases mainly include rot disease caused by *Lecanicillium aphanocladii*, *Clonostachys rosea*, *and Aspergillus niger* (Lv et al., 2022; Fu et al., 2023; Tu et al., 2024), white mold disease caused by *Paecilomyces penicil* (He et al., 2017), cobweb disease by *Cladobotryum mycophilum* (Liu Z. et al., 2023), and stipe spot disease by *Cladosporium scabrellum* (Zhang et al., 2024). Among these, *L. aphanocladii* is an important pathogen. Its characteristic symptom is a small white mold-like injury that first appears on the surface or top of the cap. The lesion then develops to surround the cap and gradually spreads to the stalk. Finally, white mold, which is a serious rot disease with a natural incidence of 30%, softens the fruiting body (Lv et al., 2022). This not only hinders the development of *M. esculenta* cultivation but also causes massive losses to farmers and agricultural companies (Yu et al., 2022). However, thus far, research on the cultivation of *Morchella* is mostly limited to the identification of pathogens, and methods of preventing and controlling diseases have rarely been studied or applied (Xu et al., 2022; Liu and Dong, 2023). Although the use of chemical control of disease has been studied for other edible fungi, the use of antibiotics is accompanied by increase risks of soil and water pollution, threats to food safety, damage to human health, and the development of antibiotic resistance in plant pathogens (Sánchez, 2004; Grogan, 2006; El-Baky and Amara, 2021; Gea et al., 2021; Li et al., 2023).

Thus, it is clearly necessary to develop new prevention and control strategies to eliminate or reduce the incidence of *M. esculenta* disease that are cost-effective and nontoxic. Biological control of plant pathogens refers to the use of beneficial microorganisms or microbial metabolites to effectively control plant diseases (Ahmed W. Q. et al., 2022; Liu Q. et al., 2022). Plant–microbe interactions play an important role in soil and plant health (Niu et al., 2020; Jia et al., 2021). The use of biocontrol microorganisms to control plant pathogens has the advantages of being green, effective, and sustainable, and it is an environmentally friendly method for the control of agricultural diseases, making it the focus of current research (Morin, 2020; Niu et al., 2020; Zhang et al., 2020). Some of the most intensively studied biological control agents are bacteria, which can use a variety of mechanisms to limit the development of plant diseases (Ahmed W. et al., 2022). Several bacterial-based products have been registered and sold as biological pesticides (Bonaterra et al., 2022). *Bacillus*, *Pantoea*, *Streptomyces*, *Trichoderma*, *Clonostachys*, *Pseudomonas*, and *Burkholderia* spp. have been widely studied as biocontrol agents reported, and some have been commercialized (Boro et al., 2022; Lahlali et al., 2022; Khan et al., 2023; Martinez et al., 2023). In some cases, biological control agents not only limit fungal diseases but also protect plants indirectly by triggering biochemical and molecular defense responses to a wide range of pathogens and by promoting plant growth (Kohl et al., 2019; Legein et al., 2020; Elnahal et al., 2022).

At present, bacteria have been utilized for biocontrol on many species of plants, and there have been some studies in edible fungi (Carrasco and Preston, 2020). Biocontrol bacteria are used in different hosts, and there have been some studies on biological control in edible fungi. For example, bioinoculants registered for mushrooms are reduced to the use of entomopathogenic nematodes and fungi as insecticides to fight mushroom flies, and two strains commercialized as *B. subtilis* QST 713 and *B. amyloliquefaciens* MBI 600 are registered as bioinoculants to control green mold disease in mushrooms (Carrasco and Preston, 2020). However, biocontrol of *M. esculenta* have not yet been reported.

Endophytic microorganisms are highly suited for biocontrol because of their stability advantage in host tissue colonization (Fontana et al., 2021). The first purpose of this study was to investigate the use of an endophytic strain of *B. subtilis* to alleviate rot disease. The physiological and biochemical characteristics and the whole genome of *B. subtilis* A9 were analyzed in order to understand the characteristics of the strain. Through the pot experiment, to explore the best way of use, and finally applied in the field to verify its biocontrol effect. Since the biocontrol mechanism of *B. subtilis* A9 is not clear, we further study the biocontrol mode of *B. subtilis* A9 through SEM observation and transcriptome analysis. Meanwhile, some evidence was provided from the point of view of transcriptome. This study has provided insight into the biocontrol mechanism of *B. subtilis*, and revealed its potential as a beneficial high-efficiency biocontrol agent.

## 2 Materials and methods

### 2.1 *Morchella esculenta* and pathogens

*Morchella esculenta* was sampled from a planting site in Jiaxing Zhejiang in March 2021. The whole fruiting body was collected, placed in a sterile bag, and brought back to the laboratory. The mycelium was isolated and cultured in potato dextrose agar (PDA; 200 g·L^−1^ potato extract, 20 g·L^−1^ glucose, and 15 g·L^−1^ agar) and grown at 28°C.

The *Lecanicillium aphanocladii* strains used for the present study were cultured in potato dextrose agar (PDA; 200 g·L^−1^ potato extract, 20 g·L^−1^ glucose, and 15 g·L^−1^ agar) and grown at 28°C (Lv et al., 2022).

### 2.2 Isolation and identification endophytic bacteria

Fresh *M. esculenta* was rinsed with sterile water for approximately 5 min. Excess water on the surface was absorbed with filter paper. Samples were washed twice with sterile water, soaked in 75% alcohol for 3 min, washed with sterile water 3 times, soaked in 3% sodium hypochlorite for 3 min, and finally washed with sterile water 4 times. The last cleaning solution was plated on medium as a control. The disinfected *M. esculenta* epidermis was peeled off with a sterile scalpel. To isolate the endophytic bacteria the tissue samples were ground using a mortar and pestle using an appropriate amount of disinfected sand and sterile water. After thorough grinding, the supernatant was smeared on NA and PDA medium plates and cultured in incubators at 37 and 28°C, and three repeats were set up. During culturing, the bacteria and fungi were observed every day, and novel isolates were purified using fresh solid medium. The purified endophytic bacteria, endophytic actinomycetes, and endophytic fungi of *M. esculenta* were transferred to NB slopes, Gaoshi No. 1 slopes, and PDA slopes, respectively, and stored in a refrigerator at 4°C (Shweta et al., 2013; Maliehe et al., 2020; Wang et al., 2020).

### 2.3 Antibiosis test

Antibiosis against *L. aphanocladii* was screened as follows. The endophytic strains were activated on NA plates, and for each one a single colony was used to inoculate NA medium and cultured at 28°C for 24 h. The *L. aphanocladii* G1 were activated on a PDA plate and cultured at 25°C for 7 days. The *L. aphanocladii* G1 and antagonistic bacteria were symmetrically spread at equal distances. A 5-mm plug of *L. aphanocladii* G1 was used to inoculate the center of the solid PDA medium, and the endophytic bacteria were used to inoculate the four corners of the plate center at a distance of 30 mm. Each bacterial strain was applied in a ring and cultured at 28°C for 10 days. The pathogen plate was used as the control to observe the growth of the colony on the plate. The inhibition rate Formula (1) (Liu Y. et al., 2022) of the plate confrontation test was as follows:


(1) $$\begin{array}{l} \mathrm{Relative}\;\mathrm{inhibition}\;\mathrm{rate}\left(\%\right)=\\ \frac{\left(\mathrm{Colony}\;\mathrm{diameter}\;\mathrm{control}-\mathrm{Colony}\;\mathrm{diameter}\;\mathrm{of}\;\mathrm{treatment}\right)\times 100}{\mathrm{Colony}\;\mathrm{diameter}\;\mathrm{of}\;\mathrm{control}} \end{array}$$

### 2.4 Screening and identification of antagonistic endophytes

First, the strain was morphologically identified. After the coated plate was cultured for 24 h, colony size, color, shape, edge, and transparency were observed by naked eye and recorded (Table 1).

**Table 1:** List of abbreviations.

Abbreviation	Full name
SEM	Scanning Electron Microscope
DEGs	Differentially Expressed Genes
GO	Gene Ontology
KEGG	Kyoto Encyclopedia of Genes and Genomes
MAPK	Mitogen-Activated Protein Kinases
ABC	ATP-Binding Cassette
PPO	Polyphenol Oxidase
SOD	Superoxide Dismutase
PAL	Phenylalanine Ammonia Lyase
CAT	Catalase
BP	Biological Process
MF	Molecular Function
CC	Cellular Component
PPP	Pentose phosphate pathway
N	Nitrogen

*Bacillus subtilis* A9 was then identified using molecular means. *B. subtilis* A9 was cultured until reaching the logarithmic growth phase. Genomic DNA was extracted using a bacterial DNA kit (Radix Scutellariae). The concentration of genomic DNA extracted from *B. subtilis* A9 was determined using the Epoch2 enzyme labeling instrument (BioTek, USA). The samples were then tested by Shenggong Bioengineering (Shanghai, China) for testing.

To further explore the physiological and biochemical properties indexes of *B. subtilis* A9, HBIG14 biochemical identification paper was used. Colonies were selected, used to inoculate a nutrient agar slope or plate, cultured at 36 ± 1°C for 24 h, and then transferred to a biochemical identification strip. If the reagent strip required a liquid test sample, a bacterial suspension method was used; if solid content was required, a zigzag line inoculation or puncture inoculation was used. After inoculation, the test strip was marked, covered, placed into the base, and cultured at 36 ± 1°C. A sealed test tube was used for anaerobic growth. After culture, the result was recorded.

### 2.5 Genome sequencing

After confirming this basic information, the entire *B. subtilis* A9 genome was sequenced. The NucleoBond^®^ HMW DNA kit (MN NucleoBond, Germany, 740160.20) was used for high-quality genome extraction from samples. DNA concentration and purity was determined using Qubit4.0 (Thermo, Q33226) and Nanodrop (SMA4000, Taiwan, China). DNA integrity was assessed using 0.75% agarose gel electrophoresis. The gDNA was separated into two parts. One was randomly fragmented to build a library with an insertion size of 300 bp. The library was assembled on an Illumina NovaSeq 6000 platform with a paired-end 150 bp sequencing strategy. Another was performed through end-repair, 3′ adenylation, and adapter and motor protein ligations. The product was purified using Agencourt AMPure XP Beads (Beckman, A63881). Finally, fragments larger than 1 KB were screened using Single-molecule nanopore DNA sequencing on MinION Flow Cell (ONT, R9.4.1).

The raw reads were filtered and assembled using Canu with default parameters. The genomic sequences were proofread using nextpolish (v1.4.1) and Pilon (v1.18). Gene prediction and annotation were generated using Prokka (Version 1.10) and the National Center for Biotechnology Information (NCBI) nr database. The functional annotation was based on protein-coding genes using the Cluster of Orthologous Groups of Proteins (COG) (Tatusov et al., 2000) and the Kyoto Encyclopedia of Genes and Genomes (KEGG) (Kanehisa et al., 2023) database. The Comprehensive Antibiotic Resistance Database^1^ was queried to predict virulence genes and antibiotic resistance genes.

The complete chromosome sequence of *B. subtilis* A9 was deposited in GenBank under accession number CP136257. The sequencing data were saved as FASTQ files and deposited to the National Center for Biotechnology Information (NCBI) under BioSample accession number SAMN37765015.

### 2.6 Scanning electron microscope (SEM) observations

To assess possible effects caused by the antagonistic bacteria on the pathogen mycelium, the mycelium was microscopically observed at the border of the growth zone in co-culture (TM4000 5 kV 11.5 mm X2.00k Mix M). Damage or hyphal changes caused by the antagonistic bacterial isolate were described by referring to the morphology of the pathogen grown in the absence of bacteria.

### 2.7 Pot and field assays

**Figure 1:**
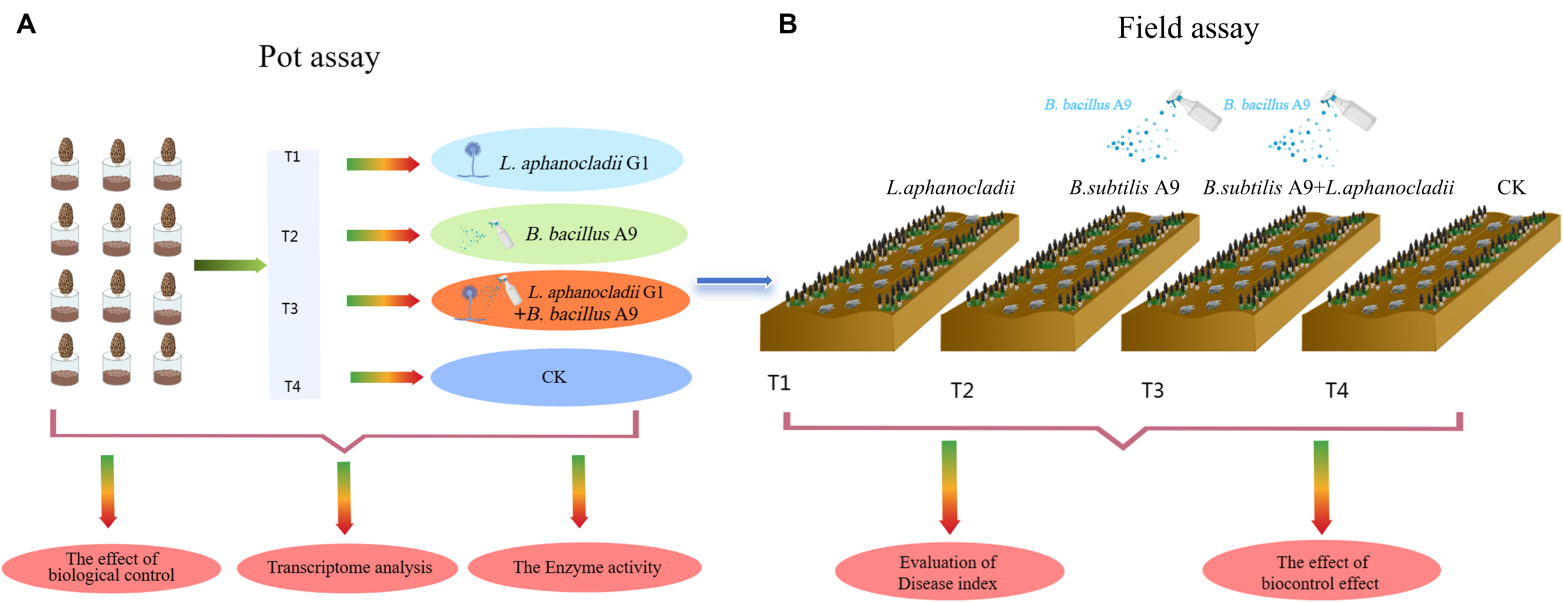
**(A)** Identity of endophytic bacteria. The morphological characteristics are shown, and 25 different bacterial species were identified. **(B)** Without inoculation of *B. subtilis* A9, *Lecanicillium aphanocladii* overgrew the PDA. In the presence of *B. subtilis* A9, the radial growth of *L. aphanocladii* was significantly inhibited, showing a clear inhibition zone between the fungal colony and the bacterial colony. **(C)** Observation of biocontrol bacteria *B. subtilis* A9 under SEM (TM4000 20 kV 12.0 mm X8.00k Mix M), a rod-shaped bacteria of 10 μ. **(D)** Four *M. esculenta* endophytes, showing stronger antagonistic activity *in vitro* against *L. aphanocladii.*

A pot assay was (Figure 1A) conducted in a growth chamber to assess the biocontrol efficacy of *B. subtilis* in reducing fungal load. The experiment was repeated three times and comprised four treatments (T1–T4):

Treatment 1: *M. esculenta* inoculated with pathogen *L. aphanocladii* G1;

Treatment 2: 1 mL 2 × 10^9^ CFU/mL *B. bacillus* A9 fermentation liquid applied to *M. esculenta* with pathogen *L. aphanocladii* G1 applied to *M. esculenta* the next day;

Treatment 3: 1 mL 2 × 10^9^ CFU/mL *B. bacillus* A9 fermentation liquid applied to *M. esculenta*;

Treatment 4: noninoculated control.

After all experiments were performed, infected *M. esculenta* and soil were autoclaved before waste disposal to avoid the spread of infection.

To further study the biological control effect in the field, we also conducted field assay (Figure 1B) site at Dongzhuang Village, Liantang Town, Qingpu District, Shanghai, China. The soil of the test site was fertile, with measures for *M. esculenta* growth in place. Irrigation was convenient. The experiment adopted a random block distribution and was divided into four plots, the area of each block is 1 m × 20 m (Supplementary Figure S1). The following four groups were used for this experiment (T1–T4):

Treatment 1: *M. esculenta* inoculated with pathogen *L. aphanocladii* G1;

Treatment 2: 100 mL 2 × 10^9^ CFU/mL *B. bacillus* A9 fermentation liquid applied to *M. esculenta* with pathogen *L. aphanocladii* G1 applied to *M. esculenta* the next day;

Treatment 3: 100 mL 2 × 10^9^ CFU/mL *B. bacillus* A9 fermentation liquid applied to *M. esculenta*;

Treatment 4: noninoculated control.

After all experiments were performed, infected plants and soil were autoclaved before waste disposal to avoid the spread of infection.

Disease severity was recorded on a 0–5 scale, with 0 representing no infection and 5 denoting complete infection (Table 2). The 0–5 scale of disease severity was classified following Ma et al. (2019), Rokni and Goltapeh (2019), and Singh et al. (2021).

**Table 2:** Grading standard of *M. esculenta* fungal disease.

Disease level	Symptom description
0	No disease spots or other infection indications.
1	A few limited lesions: spots, less than 1 mm in diameter and less than 1% of total area.
2	Irregular spots dispersed: approximately 2 mm in diameter and 1–5% of total area.
3	Lesion extension: 3–4 mm in diameter, 6–20% of total area.
4	Expanded lesions: 4 mm or more in diameter, 21–50% of the total area.
5	Expanded lesions: accounting for more than 51% of the total area.

The percentage of disease incidence was determined using the following Formula (2) (Chen et al., 2022):


(2) $$\begin{array}{l} \mathrm{Disease}\;\mathrm{severity}\;\mathrm{index}\left(\%\right)=\\ \frac{\sum \mathrm{Disease}\;\mathrm{level}\times \mathrm{number}\;\mathrm{of}\;\mathrm{Morchella}\;\mathrm{infected}}{\mathrm{highest}\;\mathrm{rating}}\\ \times \;\mathrm{total}\;\mathrm{number}\;\mathrm{of}\;\mathrm{Morchella}\times 100 \end{array}$$

Both the preventive and curative effects were calculated using the following Formula (3) (Chen et al., 2022):


(3) $$\begin{array}{l} \mathrm{Control}\;\mathrm{effect}\left(\%\right)=\\ \sum \frac{\mathrm{blank}\;\mathrm{group}\;\mathrm{disease}\;\mathrm{index}-\mathrm{treatment}\;\mathrm{group}\;\mathrm{disease}\;\mathrm{index}}{\mathrm{blank}\;\mathrm{group}\;\mathrm{disease}\;\mathrm{index}}\times 100 \end{array}$$

### 2.8 Construction and sequencing of the transcriptome

Gene expression profiles were evaluated for Treatment 3 (application of only *B. bacillus* A9 to *M. esculenta*) and Treatment 4 (noninoculated control) in pot experiments. Three biological replicates for each sample were analyzed.

Total RNA was extracted from the whole *M. esculenta* fruiting body using Trizol Reagent (Invitrogen Life Technologies), according to the manufacturer’s protocols. Quality and integrity were determined using a NanoDrop spectrophotometer (Thermo Scientific). Three micrograms of RNA were used as input material for the RNA sample preparations. RNA libraries were constructed and size-selected using the NEBNext Ultra II RNA Library Prep Kit for Illumina (New England Biolabs Inc., Ipswich, MA, USA) and the AMPure XP system (Beckman Coulter, Beverly, CA, USA), respectively. The libraries were amplified by PCR (15 cycles) and sequenced on the NovaSeq 6,000 platform (Illumina) using Shanghai Personal Biotechnology Cp. Ltd.

Raw sequencing reads of Fastq files were filtered using Fastp (0.22.0), which masked bases with Q-scores <20 and the sequence of the removed 3′ end adapters. All subsequent analyses were high-quality analysis based on Cleandata. The reference genome and gene annotation files were downloaded from the genome website. The filtered reads were mapped to the reference genome using HISAT2 (v2.1.0). HTSeq (v0.9.1) was used to statistically compare the read count values for each gene as the original expression of the gene and to standardize the expression using FPKM. The differential expression of genes was then analyzed using DESeq (v1.38.3) with the following screening conditions: expression difference multiple |log2FoldChange| > 1 and significant *p*-value <0.05.

All genes were mapped to terms for the Gene Ontology (GO) database, and the number of differentially enriched genes in each term was calculated. Top GO (v2.50.0) was used to perform GO enrichment analysis on the differentially expressed genes (all DEGs/up DEGs/down DEGs), and the *p*-value was calculated using the hypergeometric distribution method (the standard of significant enrichment was p-value <0.05). The GO terms of significant DEGs were identified to determine their main biological functions. ClusterProfiler (v4.6.0) software was used to carry out enrichment analysis of the KEGG pathways of DEGs, focusing on significant enrichment pathways with a *p*-value <0.05. The Gene Set Enrichment Analysis (GSEA) (v4.1.0) tool was used for the GSEA enrichment analysis of all genes, and a GSEA enrichment analysis pathway map was drawn.

Based on the existing reference genome,^2^ StringTie (v2.2.1) software was used to assemble the mapped reads, and the splicing results were compared with the known transcripts to obtain annotations or transcripts of information.

### 2.9 Antioxidant enzymes analysis

To confirm the transcriptome results, *M. esculenta* samples (1.0 g) were tested. The phenylalanine ammonia-lyase (PAL), catalase (CAT), peroxidase (POD), and superoxide dismutase (SOD) activities of the parental lines were determined using assay kits as directed by the manufacturers (Suzhou Keming Biotechnology Co., Ltd.). Three biological repeats of each sample were measured.

### 2.10 Data treatment and statistical analysis

The diameters of the colonies were expressed as mean values ± standard deviation (SD). All assays were repeated in at least three separate experiments. All experimental data were expressed as mean ± SD. Analysis of variance (ANOVA) was completed using the Statistical Package for GraphPad Prism 8 (v8.0.1.244). One-way ANOVA followed by *post hoc* analysis was used to compare mean values among treatments at the 5% level of significance (*p* ≤ 0.05).

## 3 Results

### 3.1 Identification of 122 isolated of endophytic bacteria

**Figure 2:**
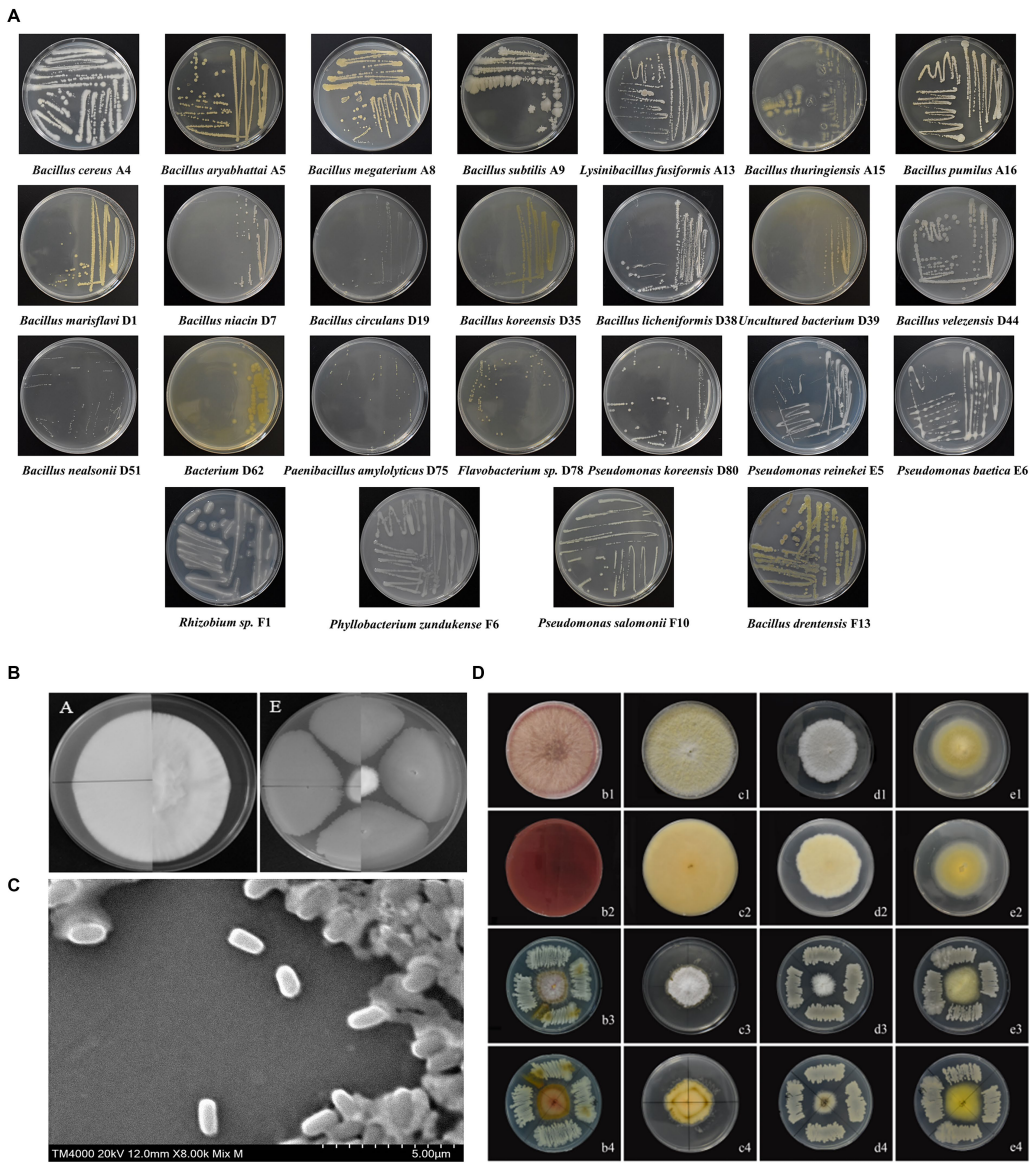
A flow chart of the pot and field assay.

A total of 122 endophytic bacteria colonies and 2 endophytic fungi colonies were isolated from *M. esculenta* and identified by 16SrDNA gene amplification. They are belonging to 25 bacteria and 2 fungi. The bacteria that were further studied were designated as A4, A5, A8, A9, A13, A15, A16, D1, D7, D19, D35, D38, D39, D44, D51, D62, D75, D78, D80, E5, E6, F1, F6, F10, and F13. For the endophytic bacteria, most of the colonies were orbicular, a few are irregular, most are white, and a few are yellow or light brown. The morphological characteristics of the bacteria are shown in Table 3, and details on their identification are shown in Figure 2A. The largest group among all the identified strains was *Bacillus*, with 15 strains, and the second largest group was *Pseudomonas*.

**Table 3:** The morphological characteristics of endophytic bacteria.

Strain code	Colony morphology			
	Form	Color	Edge condition	Surface morphology
A4	Orbicular	Milky white	Serrated	Dull, rough, opaque, waxy, flat
A5	Orbicular	Yellow	Complete	Shiny, rough, opaque, easy to provoke, flat
A8	Orbicular	Yellow	Complete	Shiny, rough, opaque, easy to provoke, flat
A9	Irregular	Milky white	Serrated	Dull, rough, opaque, waxy, flat
A13	Orbicular	Light brown	Complete	Dull, rough, opaque, waxy, flat
A15	Irregular	Yellow	Complete	Shiny, smooth, opaque, sticky, convex
A16	Orbicular	Milky white	Undulating	Shiny, smooth, opaque, easy to provoke, flat
D1	Orbicular	Yellow	Complete	Shiny, smooth, opaque, waxy, flat
D7	Orbicular	Light brown	Complete	Shiny, smooth, opaque, sticky, convex
D19	Orbicular	White	Complete	Dull, smooth, translucent, easy to provoke, convex
D35	Orbicular	Yellow	Complete	Dull, rough, opaque, waxy, flat
D38	Irregular	Milky white	Serrated	Shiny, smooth, opaque, sticky, convex
D39	Orbicular	Yellow	Complete	Shiny, smooth, opaque, easy to provoke, convex
D44	Orbicular	White	Complete	Shiny, smooth, opaque, easy to provoke, convex
D51	Orbicular	White	Complete	Shiny, smooth, opaque, easy to provoke, convex
D62	Orbicular	Yellow	Complete	Shiny, smooth, opaque, sticky, convex
D75	Orbicular	Light yellow	Complete	Shiny, smooth, opaque, easy to provoke, convex
D78	Orbicular	Yellow	Complete	Shiny, smooth, opaque, easy to provoke, convex
D80	Orbicular	White	Complete	Shiny, smooth, opaque, easy to provoke, convex
E5	Orbicular	White	Complete	Shiny, smooth, opaque, sticky, convex
E6	Orbicular	White	Complete	Shiny, smooth, opaque, sticky, convex
F1	Irregular	Milky white	Complete	Shiny, smooth, translucent, sticky, convex
F6	Orbicular	Milky white	Complete	Shiny, smooth, translucent, sticky, convex
F10	Irregular	Light yellow	Serrated	Dull, rough, opaque, waxy, flat
F13	Orbicular	Yellow	Complete	Dull, rough, opaque, waxy, flat

122 strains of endophytic bacteria were identified as belonging to 25 species of bacteria, and the code of each specie of bacteria was listed in the table.

### 3.2 General biological characteristics and genomic features of *Bacillus subtilis* A9

Among the endophytic bacteria isolated from *M. esculenta*, nine showed stronger (IR ≥ 60%) antagonistic activity against *L. aphanocladii* by *in vitro* assays. Among the nine strains, *B. subtilis* A9 was most effective in suppressing the radial growth of *L. aphanocladii* hyphae with 72% IR (Figure 2B). *B. subtilis* A9 was deposited at the Chinese typical Culture Preservation Center (CCTCC), under the accession number CCTCCNO:M20221704. Additionally, as shown in Figure 2D, *B. subtilis* A9 also exhibited significant antagonistic activity against four other pathogenic fungi, which also cause destructive diseases of *M. esculenta*, indicating that *B. subtilis* A9 has broad-spectrum activity and great potential in the control of *M. esculenta* fungal diseases.

*Bacillus subtilis* A9 is a rod-shaped Gram-positive bacteria with a length of 10 μm. (Figure 2C), present singly or in pairs, with a central spore. On NA, *B. subtilis* A9 formed creamy white and surface rough colonies with irregular edges. Strain A9 was chemo-organotrophic, D-xylose, hydrolysis D-mannitol, gelatin, liquefied starch, nitrate reduction and L-arabinose were positive (Table 4).

**Table 4:** Physicochemical properties of *B. subtilis* A9.

Biochemical reaction	V-P test	Citrate	Propionate	D-xylose	D-xylose
Result	−	−	−	+	+
Biochemical reaction	HydrolysisD-mannitol	Gelatin	Liquefied starch	pH5.7	Nitrate reduction
Result	+	+	+	−	+

**Figure 3:**
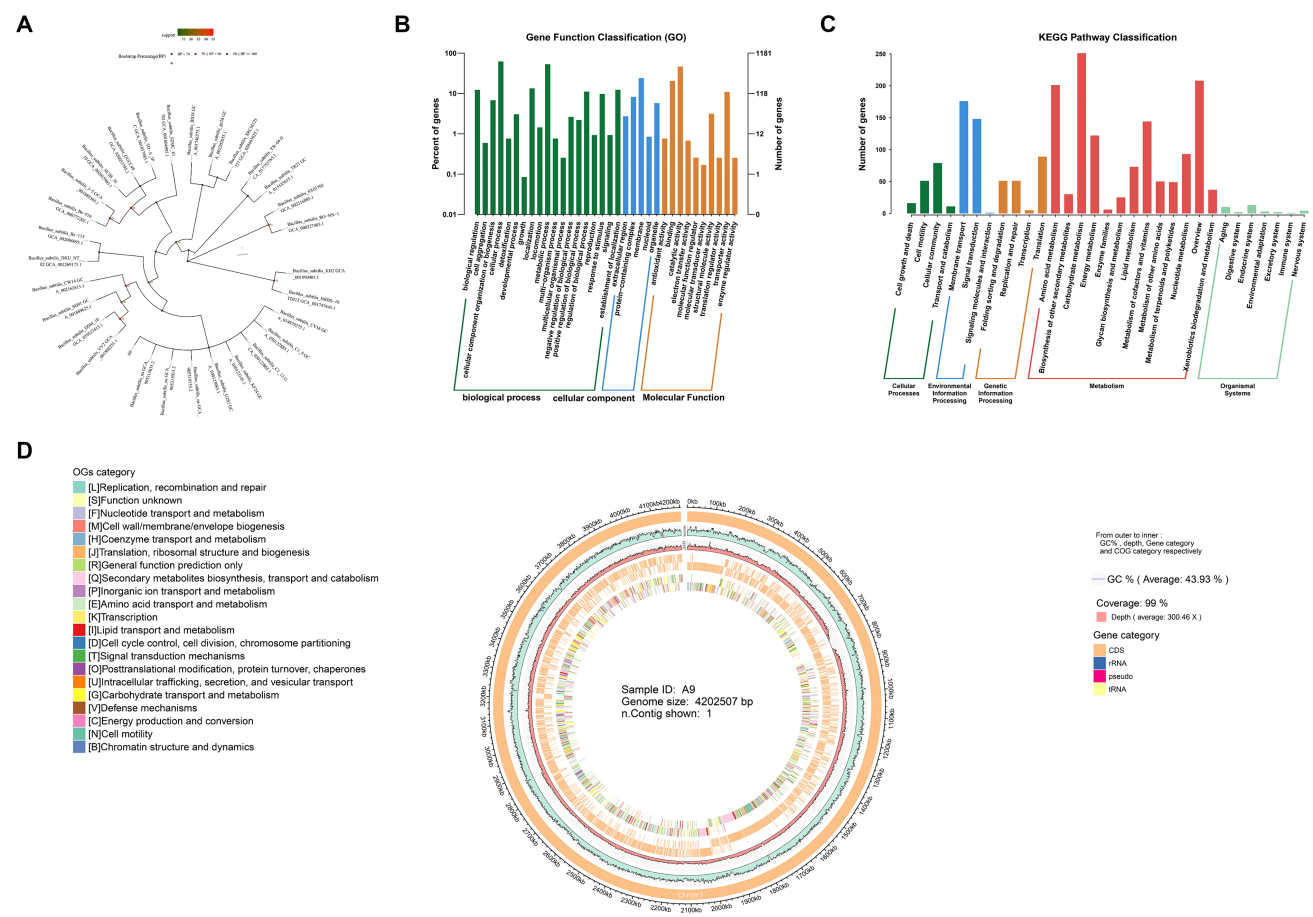
Sequencing of the whole genome of *B. subtilis* A9. **(A)** Phylogenetic tree. **(B)** GO annotations distribute bar charts. The horizontal axis is the secondary classification of GO, and the vertical axis is the number of genes in the classification (right). Percentage indicates is the total number of annotated genes (left). Different colors represent different orthologs. **(C)** KEGG pathway classification bar chart. The horizontal axis is the name of the metabolic pathway involved, and the vertical axis is the number of genes annotated to that pathway. **(D)** Genomic circos map; displayed in the circle map are GC content, sequencing depth, gene element display, and COG function display, from outside to inside.

*Bacillus subtilis* is a widely studied biocontrol species, but thus far, no *B. subtilis* has been isolated from *M. esculenta* (NCBI data). Here, we describe the complete genome sequence and annotation. A phylogenetic tree was constructed using the FastTree program based on multiple sequence alignment and cutting using mafft software, which showed a distinct branch containing the A9 strain and *Bacillus* sp. (Figure 3A).

The total size of the genome was 4,202,507 bp with a GC content of 44%. A total of 114 RNAs, including 27 rRNAs and 87 tRNAs, were identified. The total number of genes (CDS) of the *B. subtilis* A9 strain was 4,075. Of these, 2,805 were assigned a COG number. The most abundant COG category was “General function prediction only” (350 proteins), followed by “Function unknown” (313 proteins), “Amino acid transport and metabolism” (279 proteins), “Transcription” (267 proteins), “Carbohydrate transport and metabolism” (237 proteins), and “Cell wall/membrane/envelope biogenesis” (194 proteins). Circos software was used to display the genome, including its GC content, sequencing depth, gene element content, and COG function display (Figure 3D). A total of 45 carbohydrate enzyme family genes were annotated. Among these, 17 glycoside hydrolase genes were found, accounting for 37.7% of the total, and 11 glycosyl transferases, accounting for 24.4%. *B. subtilis* A9 may act by producing glycoside hydrolase and glycosyl transferase (Supplementary Figure S2).

The predicted proteins sequences were compared with KEGG and other functional databases by BLASTX, and gene function analysis was annotated using Blast 2 GO software. Among them, there were 32,053 GO annotation genes, of which 3,785 were related to the cellular component (CC), 7,150 were related to molecular function (MF), and 21,118 were related to biological process (BP) (Figure 3B). KEGG annotated 2,458 genes, including 5 levels of signal pathways, with 164 genes related to Cellular Processes, 1,683 genes related to Metabolism, 330 genes related to Environmental Information Processing, 226 genes related to Genetic Information Processing, and 55 genes related to Organismal Systems (Figure 3C).

We then focused on genes with potential roles in antibiotic production. The secondary metabolites produced by *B. subtilis* A9 were predicted with the bacterial version of AntiSMASH,^3^ and 13 genes were detected in the genome of *B. subtilis* A9 (Table 5). Among the gene clusters related to secondary metabolite synthesis identified. Among them, the known antibiotic gene clusters with high similarity were clusters for production of *fengycin*, *bacilaene*, *subtilin*, *bacillibactin, subtilosin* and *bacilysin*, with a similarity of 100% (Supplementary Figure S3).

**Table 5:** The secondary metabolites cluster by *B. subtilis* A9.

Region	Type	From	To	Most similar known cluster	Similarity
Region 1	NRPS	360,996	424,430	surfactin	82%
Region 2	lanthipeptide-class-i	1,767,989	1,794,097	–	100%
Region 3	transATPKS,PKS-like,T3PKS,NRPS	1,816,364	1,921,603	bacillaene	–
Region 4	NRPS,betalactone	1,994,208	2,071,170	fengycin	100%
Region 5	terpene	2,141,743	2,163,641	–	–
Region 6	T3PKS	2,211,355	2,252,452	–	–
Region 7	NRPS	3,233,422	3,280,558	bacillibactin	100%
Region 8	lanthipeptide-class-i	3,431,446	3,457,671	Subtilin	100%
Region 9	CDPS	3,586,386	3,607,132	–	–
Region 10	sactipeptide	3,831,858	3,853,469	subtilosin	100%
Region 11	other	3,856,461	3,897,879	bacilysin	100%
Region 12	RiPP-like	4,086,497	4,099,228	–	–
Region 13	epipeptide	4,114,416	4,136,114	thailanstatin	10%

– indicates that there is no match to similar known cluster; − indicates that similarity <10%.

### 3.3 Inhibition of *Bacillus subtilis* A9 on mycelia and spores of *Lecanicillium aphanocladii*

**Figure 4:**
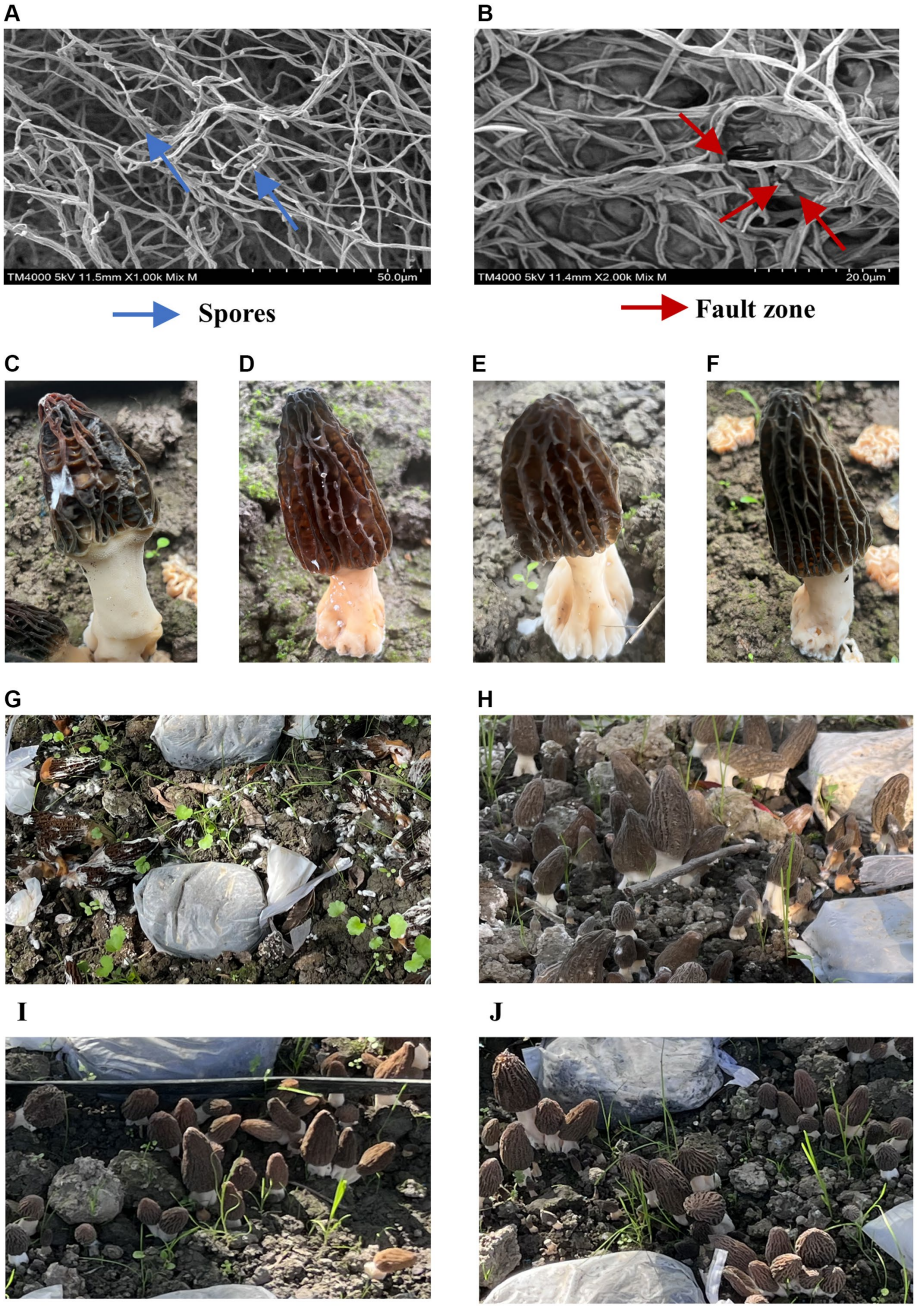
Observation of *B. subtilis* A9 with *L. aphanocladii* G1 using SEM. In **(A)**, the hyphae of the CK group were intact and uniformly distributed, and there were noticeable spores; in **(B)**, the hyphae were clearly broken and entangled after *B. subtilis* A9 treatment, and the number of spores was clearly less. **(C–F)** Pot assay observations. **(C)** *Morchella esculenta* inoculated with *L. aphanocladii* G1. **(D)** *B. subtilis* A9 was administered before *L. aphanocladii* G1. **(E)** *M. esculenta* inoculated only with *B. subtilis* A9. **(F)** Noninoculated control. **(G–J)** Field assay observation. **(G)** *M. esculenta* inoculated with pathogen G1. **(H)** *B. subtilis* A9 administered before *L. aphanocladii* G1. **(I)** *M. esculenta* inoculated only with pathogen *L. aphanocladii* G1. **(J)** Noninoculated control.

SEM observation showed that untreated *L. aphanocladii* G1 mycelium had uniform thickness and a smooth and complete surface (Figure 4A), while the hyphae showed branching, uneven thickness, and fractures in the presence of *B. subtilis* A9 (Figure 4B). In addition, the untreated hyphae was loose, while most of the treated hyphae exhibited a winding phenotype, which may have resulted from distortion of the mycelial cell wall structure. Microscopic observation revealed that the untreated mycelium produced significantly more spores than the treated group, suggesting that *B. subtilis* A9 can inhibit spore formation. In summary, co-cultivation of *L. aphanocladii* G1 with *B. subtilis* A9 is associated with abnormal mycelial development, compromised mycelial integrity, and decreased spore formation.

### 3.4 Biocontrol of *Bacillus subtilis* A9 against *Morchella esculenta* rot disease caused by *Lecanicillium aphanocladii*

We then conducted a pot experiment to assess the effects of *B. subtilis* A9 on *L. aphanocladii* G1 pathogenicity of *M. esculenta.* After 5 days of culture, the disease symptoms of the experimental group sprayed with *B. subtilis* A9 were significantly lighter than those of the control group (Figures 4C,D). Moreover, spraying A9 alone had no obvious effect on *M. esculenta* (Figure 4E).

Pot experiments showed that the best biocontrol was achieved if the *B. subtilis* A9 was sprayed before inoculation with the pathogen. We then used this strategy to conduct a field experiment. The results of the field experiment (Figures 4G–J) showed that the disease incidence substantially decreased when the *M. esculenta* was pre-treated with the biocontrol bacteria; the disease index for the biocontrol bacteria treatment was 31.8%, compared with 84.8% when treated with the pathogen alone. The control effect of the A9 treatment was 62.5%.

### 3.5 Transcriptomic profiles of *Morchella esculenta* influenced by *Bacillus subtilis* A9

To study the gene expression changes in *M. esculenta* after spraying with *B. subtilis* A9, the RNA libraries of *M. esculenta* treated with and without *B. subtilis* A9 were sequenced using the Illumina Hiseq platform. A total of 39.92 Gb of clean bases were generated, and the generated clean bases reached more than 6.65 Gb per clean sample. The total number of reference genomes of *M. esculenta* was more than 87.71%. The proportion of clean bases was more than 93.47% (Supplementary Table S1). More than 97.57% of these readings were clean at the Q20 level, and more than 93.38% were clean at the Q30 level. The number of reads corresponding to the measured GC content of each sequence was very close to the theoretical value. These results showed that the sequencing data were of good quality and could be used for follow-up analysis.

**Figure 5:**
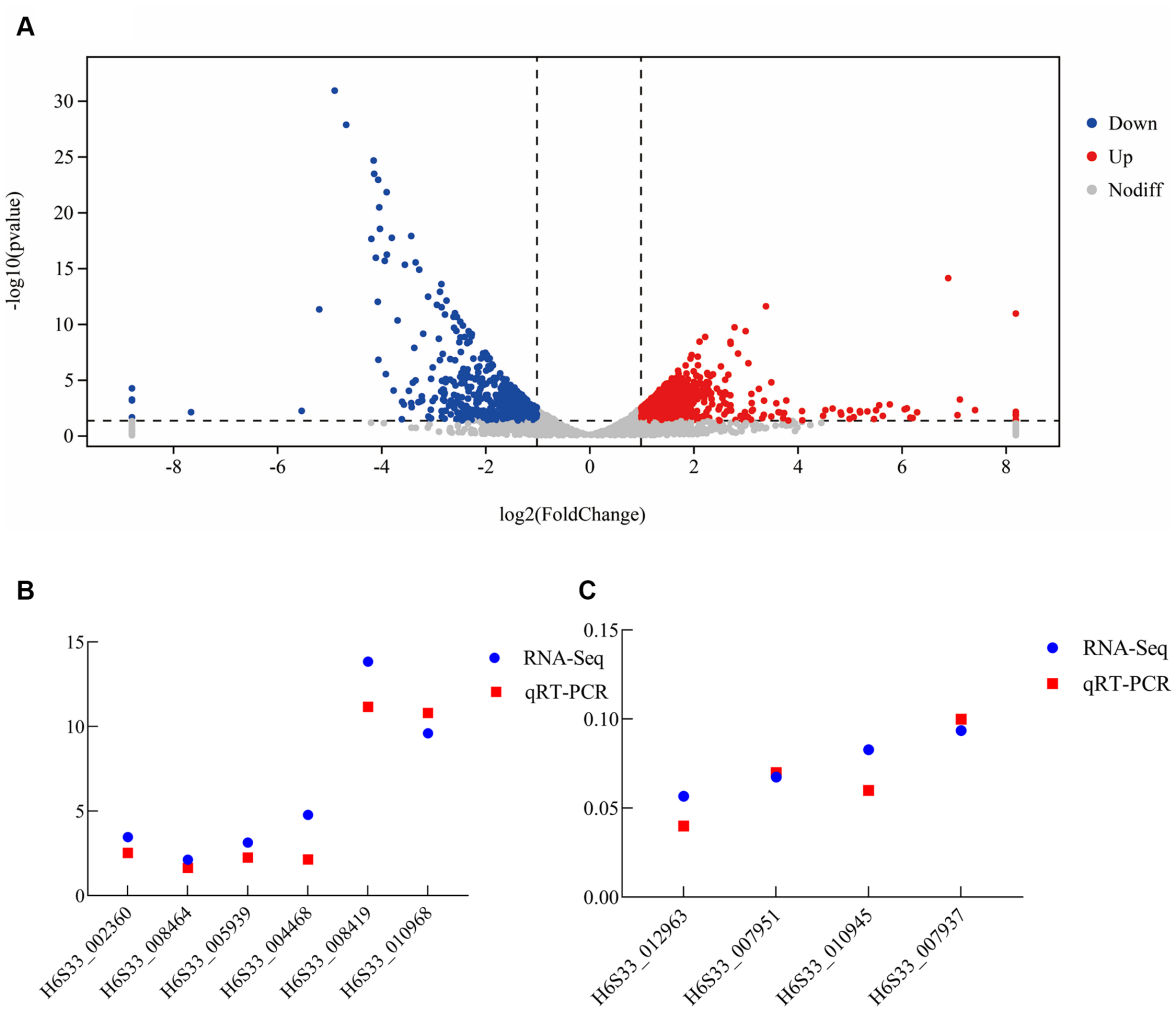
Transcriptome analysis of *B. subtilis* A9 on *M. esculenta*. **(A)** Differentially expressed gene analysis. **(B,C)** RT-qPCR verification of upregulated **(B)** and downregulated differentially expressed genes **(C)**.

Some of the upregulated (including H6S33_004468, H6S33_002360, and H6S33_005939) (Figure 5B) and downregulated DEGs (including H6S33_007951, H6S33_010945, H6S33_012963, and H6S33_007937) (Figure 5C) were selected and validated by RT-qPCR. The results showed that the actual expression of the selected DEGs was consistent with the trend of gene expression obtained by transcriptome analysis (Supplementary Table S2).

The main purpose of transcriptomics is to identify different genes between comparison groups to reveal their different molecular mechanisms. Thus, in the analysis results, the analysis of gene expression differences is the most important. Gene expression analysis of the two samples showed that there were 1,246 DEGs, of which 637 were upregulated and 609 were downregulated after the application of biocontrol strain *B. subtilis* A9 (Figure 5A). In addition, the upregulated genes showed functions, such as “oxidoreductase activity” and “response to stress,” some key genes such as peroxidase (H6S33_002360), NAD (P) H-hydrate epimerase (H6S33_008464), choline dehydrogenase (H6S33_012259) indicating that A9 may induce host Induced Systemic Resistance (ISR) by regulating the expression of genes involved in stress response and redox metabolism.

The sequencing data were saved as FASTQ files, and deposited to the National Center for Biotechnology Information (NCBI) (BioProject accessions: PRJNA1026656; BioSample accessions: SAMN37755107, SAMN37755108, SAMN37755109, SAMN37755110, SAMN37755111, SAMN37755112).

**Figure 6:**
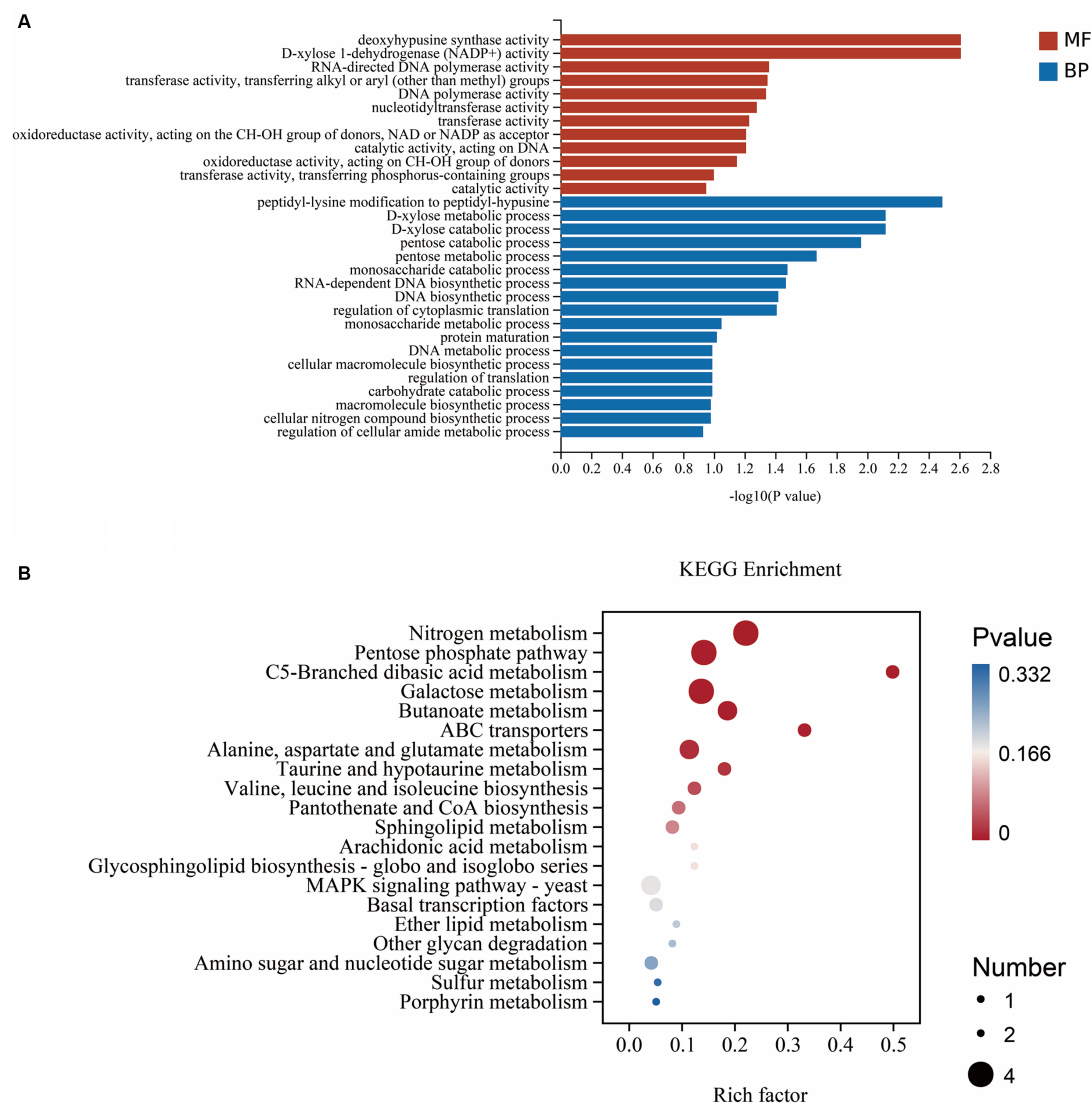
**(A)** GO enrichment. Abscissa is GO term, and ordinate is GO term enriched-log10 (*p*-value). **(B)** KEGG enrichment. The abscissa is the rich factor (the number of differential genes annotated to a pathway/the total number of genes annotated to the pathway), and the ordinate is the pathway. The size of the point in the map indicates the number of genes annotated in the corresponding pathway (upregulated or downregulated, related to the gene set selected at the time of analysis), and the color indicates the level of significance.

Gene Ontology (GO) was used to classify the functions of DEGs into three basic categories—BP, CC and MF. The number of significant genes in each item was calculated to determine the corresponding biological function of the active genes in the interaction between *B. subtilis* A9 and *L. aphanocladii* G1. The top 30 items enriched by GO showed that (Figure 6A) these genes were mainly enriched in two categories: BP and MF. The DEGs involved in the catabolic process and macromolecular synthesis process account for the majority of BP. The terms with the highest enrichment degree were peptidyl-lysine modification to peptidyl-hypusine, D-xylose metabolic process, and D-xylose catabolic process. The DEGs associated with MF involved mostly catalytic activity. The DEGs were significantly more abundant in deoxyhypusine synthase activity, D-xylose 1-dehydrogenase (NADP+) activity, transferase activity, oxidoreductase activity, acting on the CH-OH group of donors, and catalytic activity (Oliveira et al., 2020). In addition, genes associated with pyruvate dehydrogenase (H6S33_009813), DDE-type integrase/transposase/recombinase (H6S33_003558), Deoxyhypusine synthase (H6S33_009792) were also detected. In terms of MF, the expression level of D-xylose1-dehydrogenase (NADP+) was relatively high, and there were pathways related to enrichment of the antioxidant stress response. Thus, we speculated that antioxidant enzymes were involved in this process. GO analysis showed that *B. subtilis* A9 influenced *M. esculenta* by affecting its catalytic activity, catabolic process and macromolecular synthesis process (Dave et al., 2021).

To further investigate the biological pathways mediating the effects of A9 on *M. esculenta*, we performed the KEGG functional annotation analysis of all DEGs identified in RNA sequencing. The top 20 enriched KEGG pathways of DEGs are analyzed and shown in Figure 6B (the lower the *p*-value, the greater the degree of enrichment). The results showed that the DEGs participate in three pathways: metabolism, genetic information processing and environmental information processing. DEGs were mainly enriched in nitrogen metabolism pathway, Ferredoxin-dependent glutamate synthase 1FdGOGAT (H6S33_011339) and carbonic anhydrase CA (H6S33_007248) were key genes that up regulated in this pathway. The Fold Change are 10.6 and 8.4, respectively. Followed by Pentose phosphate pathway (PPP), the irreversible oxidative section of the pathway is a major source of the reducing equivalent NADPH, for biosynthesis and maintaining the redox potential necessary to protect against oxidative stress (Xiong et al., 2009). MAPK was the signaling pathways related to disease resistance (Meng and Zhang, 2013; Dahuja et al., 2021). The genes encoding catalase CAT (H6S33_001409), tRNA dihydrouridine synthase DusB (H6S33_001297) and NAD(P)-binding protein NAD(P)BP (H6S33_000823) were induced in MAPK signaling pathway. What’s more, KEGG analysis showed that A9 influenced *M. esculenta* by regulating amino acid metabolism and catabolic metabolism. These results suggested that A9 considerably influenced the gene expression and metabolic pathway of *M. esculenta*.

### 3.6 Effect of *Bacillus subtilis* A9 on activities of antioxidant enzymes in *Morchella esculenta* during biological control

**Figure 7:**
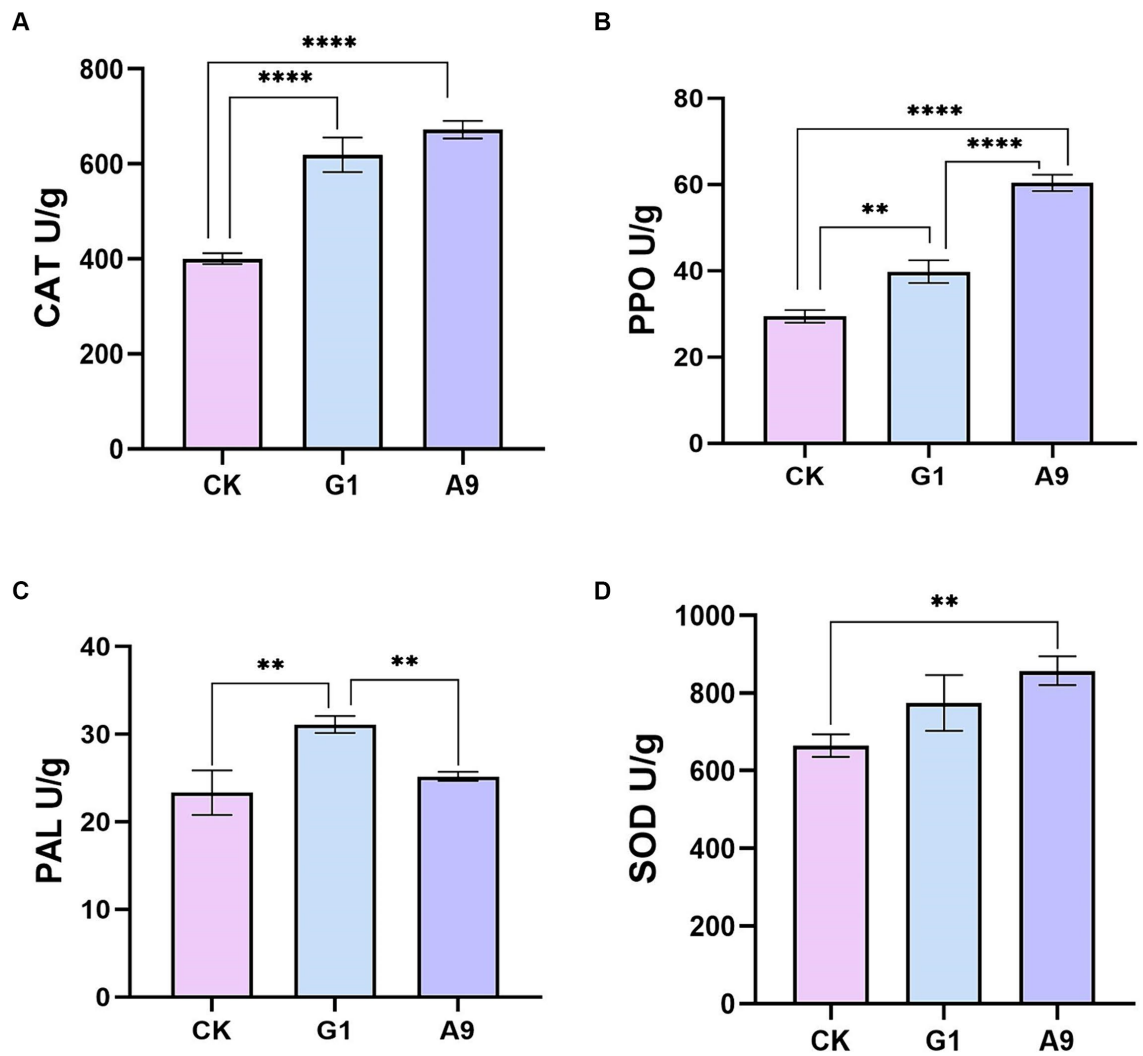
Antioxidant enzyme analysis: **(A)** catalase (CAT), **(B)** polyphenol oxidase (PPO), **(C)** phenylalanine ammonia lyase (PAL), and **(D)** superoxide dismutase (SOD). All data were presented as means of three replicates ± SD, and error bars represent SD for three replicates. Means with asterisk have significant differences(∗∗*p* < 0.01 and ∗∗∗∗*p* < 0.0001).

Four enzymes, PPO, SOD, PAL, and CAT, were used as plant disease resistance markers to assess the effects of strain *B. subtilis* A9 of *M. esculenta* infected with pathogen *L. aphanocladii* G1. The activities of PPO, SOD, PAL, and CAT were higher than in *M. esculenta* co-inoculated with *B. subtilis* A9 and *L. aphanocladii* G1 bacteria than when treated with just the pathogen, indicating that *B. subtilis* A9 enhanced *M. esculenta* defense enzyme activities (Figure 7).

## 4 Discussion

Many microorganisms have the ability to control fungal and bacterial diseases. Many of the microorganisms that have proven beneficial have been developed into products that have been commercialized. In this work, *B. subtilis* A9 isolated from *M. esculenta* was found to be effective as biocontrol bacteria. It can form spores and survive in the soil for a long time under harsh environmental conditions. Currently, several strains of *Bacillus* spp. are used as biocontrol agents against crop diseases (Bonaterra et al., 2022; Qiao et al., 2023; Idris et al., 2024). *M. esculenta* is an important edible fungus that has high economic, nutritional, and medicinal value. White mold disease in *M. esculenta* leads to a sharp decrease in yield. Due to the immature development of artificial cultivation technology, the decrease in the production of *M. esculenta* caused by rot diseases will make it even worse. Plant endophytes have been considered an excellent source of biocontrol strains and bio-inoculants in recent years due to their unique ability to thrive inside plant tissues, suppress plant pathogens, improve bacterial colonization, and promote plant growth (Chen et al., 2020). However, there are few reports of endophytes in edible fungi, and this is the first report on the endophytes of *M. esculenta* and their use as biocontrols.

In this study, 122 strains of endophytic bacteria were isolated from *M. esculenta*, of which *B. subtilis* A9 showed strong antagonism against *L. aphanocladii* G1. The greenhouse pot experiment showed that the biocontrol effect of *B. subtilis* A9 pre-treatment was better. This study demonstrated that the endophytic bacteria of *M. esculenta* are excellent as candidates for biocontrol. The use of these endophytic bacteria provides a feasible and ecologically friendly choice for the control of *M. esculenta* white rot disease. In our field experiment, the biocontrol effect of *B. subtilis* A9 was remarkable, resulting in a decrease in disease incidence of over 50%. *B. subtilis* has been widely studied and has been used as a biocontrol agent for several different species, and its mechanism of action is also understood to some extent. The application of *B. subtilis* JY-7-2L fermentation culture significantly reduced the severity of southern blight disease caused by *Aconitum carmichaelii* Debx by up to 30%, and the authors suggested that the possible mechanisms involved the production of hydrolytic enzymes and antimicrobial compounds (Zou et al., 2023). *B. subtilis* RSS-1 exerts a good inhibitory effect on *Phytophthora sojae* by inhibiting mycelium growth, cyst germination, and zoospore motility (Liu et al., 2019). *B. subtilis* E11 exhibited very strong antifungal ability, not only inhibiting of *Aspergillus flavus* on chili by 64% but also suppressing 81% of AFB1 at 24 h (Yuan et al., 2023). *B. subtilis* YPS-32 had a control efficacy of 84% against potato common scab. The mode of action of strain YPS-32 is thought to occur through with antimicrobial effects, such as surfactants and fengycin (Zhou et al., 2022). Many *B. subtilis* strains have been or are being registered as biological control products. Hence, *B. subtilis* has excellent potential for commercial biological control applications. However, the application of *B. subtilis* as a biocontrol bacterium in edible fungi has not been explored.

Understanding the mechanism of interaction between biocontrol bacteria and target plant pathogens is very important to strengthen the application of biocontrol bacteria in agriculture. In the past few decades, there has been a continuous understanding of the mechanism of biological control. The biocontrol mechanisms of biocontrol bacteria can now be divided into two categories. One is to inhibit the growth of pathogens directly through multiple mechanisms, such as parasitism, competition, and antibacterial activity (Fira et al., 2018; Soni and Keharia, 2021; Zhong et al., 2023). The other is to activate the expression of genes related to plant resistance to pathogens through some signal molecules, including secreted enzymes and secondary metabolites. Induce the resistance of host plants to pathogens and indirectly affect pathogens through growth promotion (Chen et al., 2022; Miftakhov et al., 2022; Yuan et al., 2022; Zhang et al., 2022). Direct and indirect biocontrol mechanisms usually cooperate to produce biocontrol effects on pathogens.

Many biocontrol bacteria can synthesize a diverse array of antibiotics, which are commonly linked by their ability to inhibit the growth of plant pathogens. Zeriouh et al. (2014) reported that the LP antibiotics *bacillomycin* and *fengycin*, synthesized by *B. velezensis* UMAF6614 acted directly to inhibit the growth of powdery mildew. To further determine the biological control mechanism of *B. subtilis* A9, we sequenced its genome. Genome-wide analysis revealed an antibiotic gene cluster, suggesting that it may exert it biocontrol effects through the production of antibiotics. Compared with the database, 13 gene clusters were discovered, and 6 of these matched known gene clusters. To the best of our knowledge, 5 of the gene clusters have not been reported. Cyclic lipopeptides from the *surfactin, iturin*, and *fengycin* families have been shown to have broad-spectrum antagonistic activities against plant pathogenic bacteria, fungi, and viruses (Fira et al., 2018; Miao et al., 2023) showed the important competitive role of *bacillaene* in *Bacillus* survival. Bacillibactin production restrained *in vitro* and in planta growth of the non-susceptible (to MBI600) pathogen *Pseudomonas syringae*pv. Tomato (Dimopoulou et al., 2021). Algburi et al. (2016) demonstrated that *subtilosin* prevented biofilm formation by inhibiting bacterial quorum sensing. The biological activity of these secondary metabolites is of great significance in the biological control of plant diseases. Hence, some of the clusters identified are associated with the production of antibiotics that have confirmed biocontrol effects on pathogenic bacteria, and several unknown gene clusters are interesting and worthy of further study. At the same time, we propose that the biocontrol ability of *B. subtilis* A9 could be enhanced by engineering the strain and to increase its production of antibiotics. To the best of our knowledge, this is the first time that *B. subtilis* has been isolated from *M. esculenta* endophytes, contributing to the improvement of the database and the further exploration of biocontrol bacteria.

We speculate that *B. subtilis* A9 acts directly on pathogen *L. aphanocladii* G1 through antibacterial metabolites. In addition, through a plate confrontation experiment in which *L. aphanocladii* G1 and *B. subtilis* A9 were co-cultured, we observed marginal hyphae under an electron microscope. The *L. aphanocladii* G1 hyphae treated with *B. subtilis* A9 showed that, compared to the control group, it produced fewer spores and featured longer hyphae that were broken and entangled. We speculate that *B. subtilis* A9 may cause adhesion of hyphae by destruction of the fungal cell wall, which may be its primary mode of action (Fernandes et al., 2021). In addition to acting directly on pathogens, A9 may also indirectly adjust the resistance of *M. esculenta* to the disease. Transcriptomic profiles shows that A9 regulates the disease resistance of *M. esculenta* by affecting gene expression.

Antioxidant defense systems are a prominent element in plant responses to environmental stress (Williamson and Scandalios, 1993). Increasing the activities of antioxidant enzymes and defense enzymes and up-regulating the expression of defense-related genes play an important role in inducing plant innate immunity, thus enhancing plant resistance to various types of stress (Gangireddygari et al., 2021). In this study, the peroxidase gene POD (H6S33_002360), NAD(P)H-hydrate epimerase gene (H6S33_008464) and the catalase CAT (H6S33_001409) involved in antioxidant enzyme were up-regulated. Antioxidant enzyme is an important part of the *M. esculenta* antioxidant system, which has the function of resisting oxidative damage (Li et al., 2016). The increased activity of catalase (CAT) and peroxidase (POD) can inhibit the accumulation of ROI (ROS intermediate oxygen intermediates) (Wagner et al., 2013), thus reducing the oxidative damage caused by oxidative stress in *M. esculenta*. Interestingly, the expression of CAT was increased in antioxidant enzyme experiments, and the effect of *B. subtilis* A9 on CAT content of *M. esculenta* was higher than that of *L. aphanocladii* G1 (Figure 7A). We speculate that A9 can induce higher levels of CAT, thereby improving the resistance of pathogenic fungi.

Nitrogen metabolism is the pathway where DEGs are most enriched. Recent results show that ROS are necessary for growth and development. Strikingly, the regulation of nitrogen (N) metabolism is closely related to ROS in response to both carbon (C) and N availability. Nitrogen (N) is one of the most important mineral nutrients required by higher plants. N metabolism can promote plant growth (Wang et al., 2014; Chaput et al., 2020). Carbonic Anhydrases CAs (H6S33_007248) is a kind of zinc-containing metalloenzymes, which plays an important role in regulating cell pH, carbon dioxide transport, electrolyte balance and cell homeostasis, and is very important for cell survival and proliferation. Carbonic anhydrase inhibitors have applied in a series of therapeutic fields (Supuran, 2018; Supuran and Capasso, 2020). Therefore, we speculate that the regulation of *B. subtilis* A9 on nitrogen metabolism of *M. esculenta* plays a key role in the process of disease resistance by regulating cell homeostasis and proliferation.

As a metabolic pathway of sugar independent of the classical EMP-TCA pathway, pentose phosphate pathway (PPP) has important biological significance, which is mainly reflected in: (1) providing reductant NADPH; for chemical reactions in organisms; (2) providing precursors for the synthesis of other chemicals in organisms; and (3) improving the disease resistance of plants (Bussell et al., 2013; Wushensky et al., 2018). DEGs mainly enriched in N metabolism, followed by PPP, so A9 was speculated that can improve the disease resistance of *M. esculenta* by inducing this pathway. The MAPK signal pathway is another important pathway enriched by DEGs. It regulates many important processes of plant growth and development, stress resistance, and disease resistance (Meng and Zhang, 2013; Manna et al., 2023). CAT (H6S33_001409) is an important upregulated gene enriched in this pathway. Catalases, naturally occurring oxidoreductases, are the major player of defense system against oxidative stress in all the three domains of life. Catalase activities in wild-type plants are likely to be a significant part of plant responses to changes in environmental conditions or biotic challenge (Mhamdi et al., 2010; Shaeer et al., 2021). These results explain the indirect disease resistance of *B. subtilis* A9 and lay a foundation for the study of its biocontrol mechanism.

Previous studies have shown that *Bacillus* has biocontrol effect on many plant pathogens. *Bacillus* strains exhibit their biocontrol capacity predominantly through inhibitory activity on the growth of plant pathogens, as well as inducing systemic resistance in plants and competing for ecological niches with plant pathogens (Fira et al., 2018; Abdelkhalek et al., 2020; Islam et al., 2022). In this study, we also revealed the action mechanism of *B. subtilis* A9, but there are few studies on the colonization of *B. subtilis* A9, so further research should be carried out. At present, there are almost no *Bacillus* acting on the diseases of edible fungi, we have found a strain of *B. subtilis* A9 which can prevent the fungal diseases of *M. esculenta* for the first time. The potential *B. subtilis* A9 is expected to be used to control the diseases of related edible fungi. The industrial production of biocontrol agents can be mainly grouped into two types: microbial pesticides and microbial fertilizers (Meng et al., 2022). Our research should develop and innovate the optimal form of *B. subtilis* A9 biocontrol agents. As a form of biological control, biocontrol bacteria have important value in reducing diseases and promoting growth. Meanwhile, biocontrol agents can protect the environment by reducing the use of chemical pesticides. However, there are still many problems restricting the development of biological control industry. For example, the basic research on the mechanism of microbial action is still insufficient, the role of biological pesticides is unstable, the form of biological control is single, and so on. The synthetic microbial community and developing different biocontrol agents carriers are key research directions for improving biocontrol efficiency.

## 5 Conclusion

In summary, we concluded that the screened endophytic *B. subtilis* A9 of *M. esculenta* significantly inhibited the occurrence of rot disease and the control effect was 62.5%. Genes related to antibiotic gene clusters were present in the genome of *B. subtilis* A9, which showed that A9 can produce secondary metabolites of antibiotics on pathogens. The way of *B. subtilis* A9 against the pathogen *L. aphanocladii* G1 was associated with abnormal mycelial development, compromised mycelial integrity, and decreased spore formation. Through transcriptome profiles analysis, the suggested biocontrol mechanism involved in *B. subtilis* A9 was to resist pathogens by regulating the activity of antioxidant enzymes and the metabolic pathways related to disease resistance of *M. esculenta*. Therefore, *B. subtilis* A9 is an ideal candidate to be developed as a biopesticide against rot disease as well as other diseases of *M. esculenta*.

This study provides theoretical and technical knowledge for the efficient use of microbial resources for biological control of agricultural diseases, which may lead to the improvement of new biocontrol agents for production. However, in the future, the tripartite interaction studies comprising the *M. esculenta*, biocontrol bacteria and the pathogens will provide new in-depth insights into the biocontrol mechanisms of *B. subtilis* A9. In addition, it is necessary to further explore the commercial form of *B. subtilis* A9 and extend the shelf-life of *B. subtilis* A9 based-formulations.

## Data availability statement

The datasets presented in this study can be found in online repositories. The names of the repository/repositories and accession number(s) can be found in the article/Supplementary material.

## Author contributions

XC: Data curation, Formal analysis, Investigation, Software, Validation, Visualization, Writing – original draft, Writing – review & editing. YZ: Investigation, Methodology, Project administration, Software, Supervision, Writing – original draft, Writing – review & editing. SC: Data curation, Investigation, Software, Validation, Visualization, Writing – original draft, Writing – review & editing. LS: Data curation, Investigation, Software, Validation, Writing – original draft, Writing – review & editing. GW: Data curation, Formal analysis, Software, Supervision, Writing – original draft, Writing – review & editing. YS: Data curation, Formal analysis, Project administration, Software, Writing – original draft, Writing – review & editing. YC: Data curation, Formal analysis, Methodology, Project administration, Resources, Supervision, Writing – original draft, Writing – review & editing. BL: Conceptualization, Funding acquisition, Methodology, Project administration, Resources, Writing – original draft, Writing – review & editing.

## Footnotes

^1^ https://card.mcmaster.ca/

^2^ https://www.ncbi.nlm.nih.gov/genome/?term=Morchella+sextelata

^3^ http://antismash.secondarymetabolites.org/
