# Supplementary material for: Biocontrol potential of endophytic Bacillus subtilis A9 against rot disease of Morchella esculenta
Source: Front Microbiol. 2024 May 30;15:1388669. doi: 10.3389/fmicb.2024.1388669 (PMC11169702; doi:10.3389/fmicb.2024.1388669)
Supplement: Supplementary file 2 [file Presentation_1.PPTX]

## Slide 1
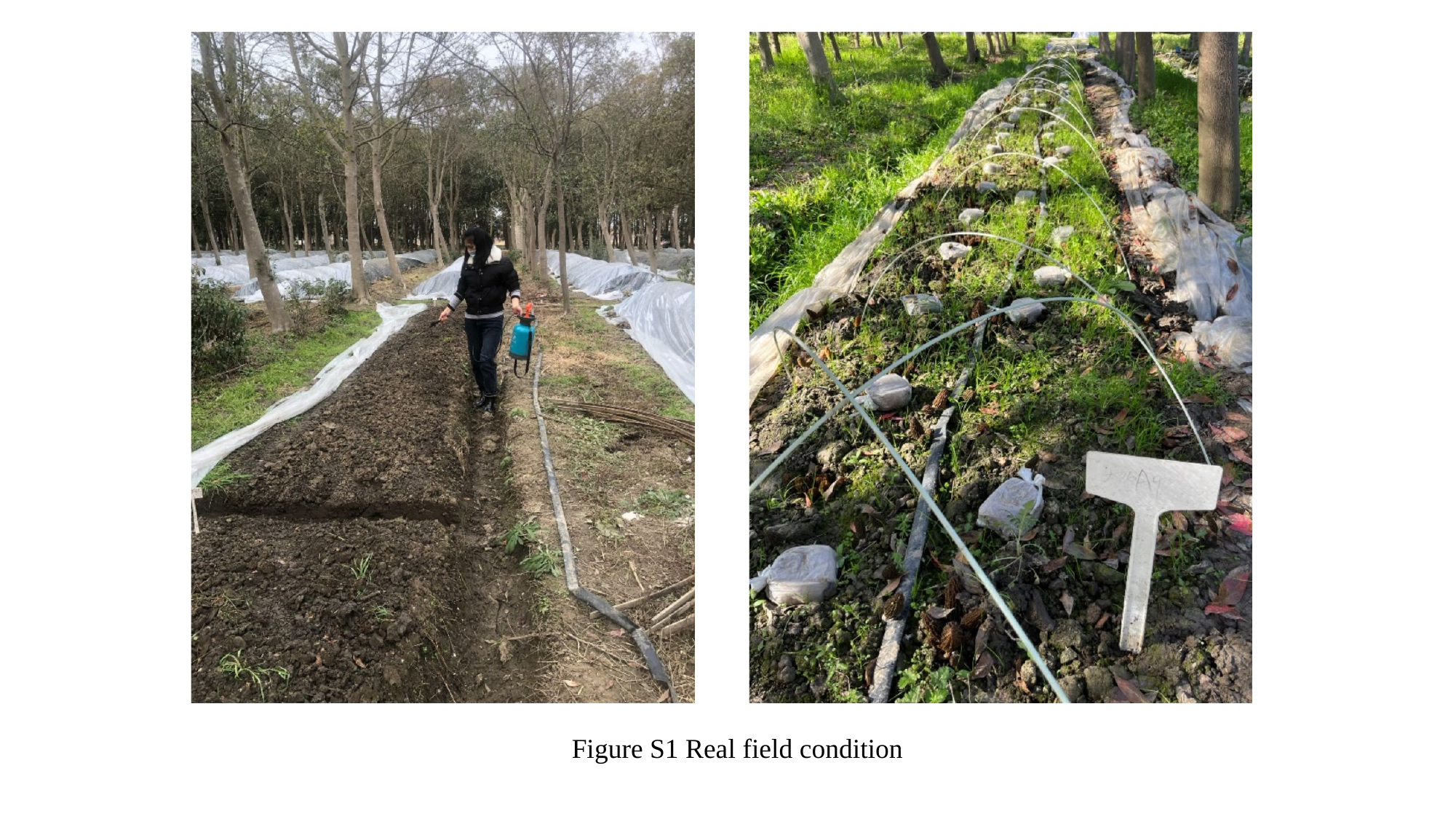

Figure S1 Real field condition

## Slide 2
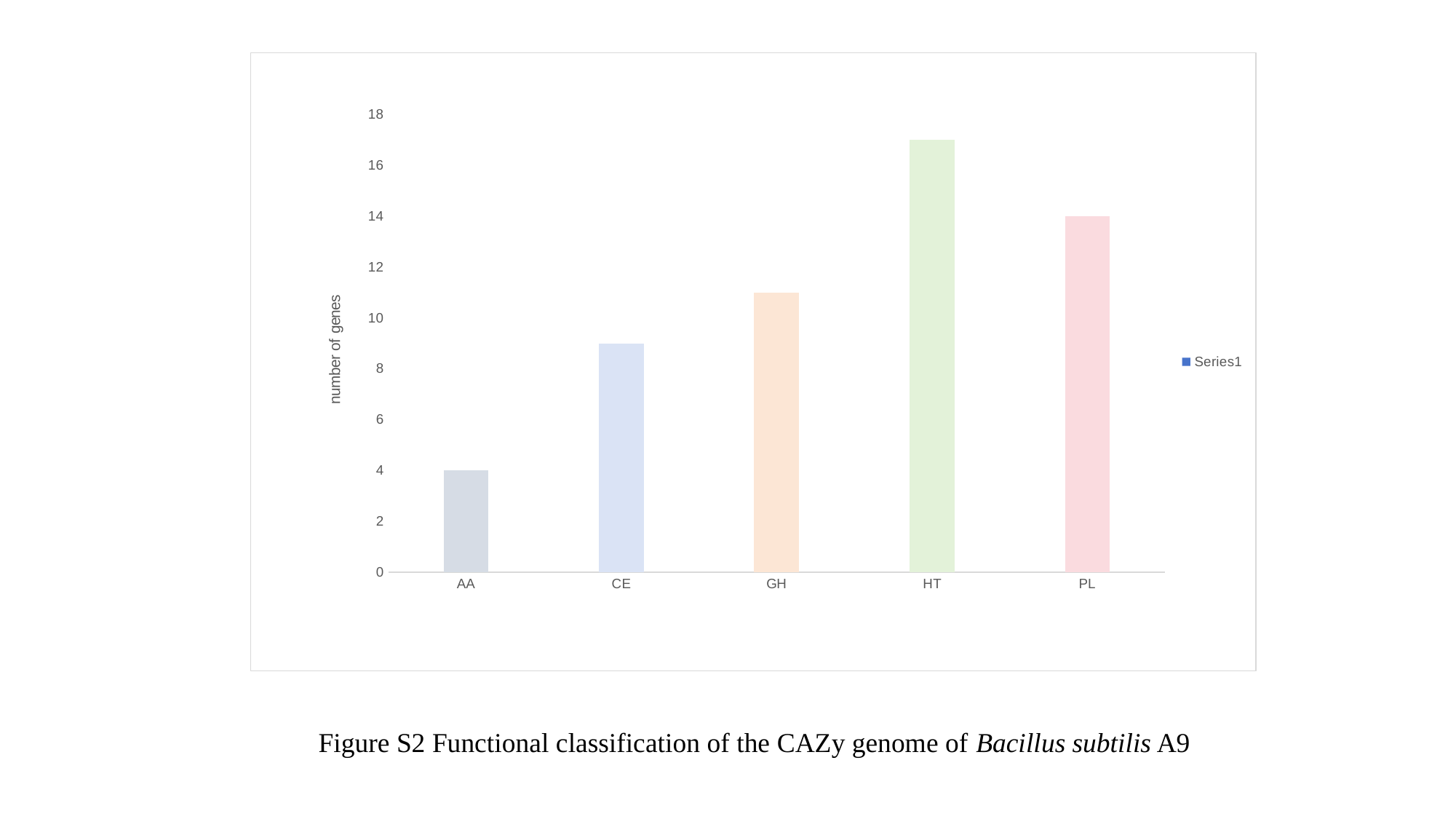

### Chart
| Category | |
|---|---|
| AA | 4.0 |
| CE | 9.0 |
| GH | 11.0 |
| HT | 17.0 |
| PL | 14.0 |Figure S2 Functional classification of the CAZy genome of Bacillus subtilis A9

## Slide 3
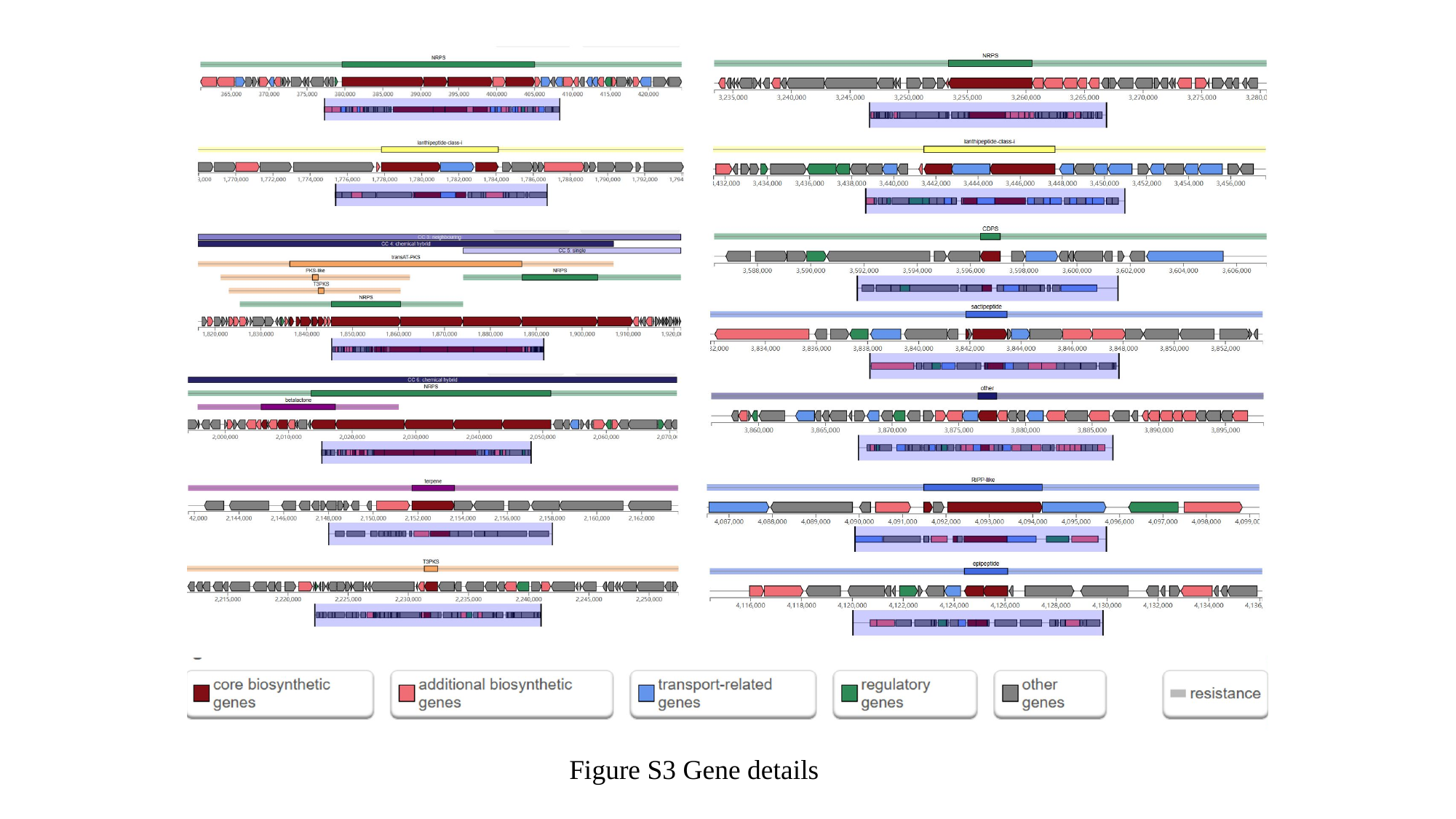

Figure S3 Gene details
